# Biomass Juncus Derived Nitrogen-Doped Porous Carbon Materials for Supercapacitor and Oxygen Reduction Reaction

**DOI:** 10.3389/fchem.2020.00226

**Published:** 2020-04-15

**Authors:** Guanghua He, Genping Yan, Yonghai Song, Li Wang

**Affiliations:** ^1^Engineering & Technology Research Center for Environmental Protection Materials and Equipment of Jiangxi Province, College of Materials and Chemical Engineering, Pingxiang University, Pingxiang, China; ^2^Key Laboratory of Functional Small Organic Molecule, Ministry of Education, Key Laboratory of Chemical Biology, Jiangxi Province, College of Chemistry and Chemical Engineering, Jiangxi Normal University, Nanchang, China

**Keywords:** juncus, nitrogen-doped porous carbon, supercapacitors, oxygen reduction reaction, renewable materials

## Abstract

Juncus is a perennial herb aquatic plant found worldwide, with high reproductive ability in warm regions. It has three-dimensional hierarchical porous triangular networks structures composited of tubular fibers. Here, juncus derived nitrogen-doped porous carbon (NDPC) was prepared by mixing juncus and ZnCl_2_ through one-step pyrolysis and activation which is a low-cost, simple, and environmentally friendly method. The NDPC had hierarchical porous structures and a high specific surface area and was applied for supercapacitor and oxygen reduction reaction (ORR). The resulted NDPC-3-800 was prepared by mixing juncus with ZnCl_2_ at a mass ratio of 1:3 and then carbonized at 800°C, it was used as electrode material of a supercapacitor. The supercapacitor exhibited excellent specific capacitance of 290.5 F g^−1^ and 175.0 F g^−1^ in alkaline electrolyte at the current densities of 0.5 A g^−1^ and 50 A g^−1^, respectively. The supercapacitor showed good cycle stability, and the capacitance was maintained at 94.5% after 10,000 cycles. The NDPC-5-800 was prepared by mixing juncus with ZnCl_2_ at a mass ratio of 1:5 and then carbonized at 800°C. It exhibited outstanding ORR catalytic activity and stability attributing to their high specific surface area and abundant actives sites. The juncus can derive various materials for application in different fields.

## Introduction

With the rapid development of human civilization, people will face an energy crisis (Lei et al., 2016; Ma Q. et al., 2017). New energy sources such as supercapacitors and fuel cells have attracted extensive attention. Supercapacitors are considered to be energy storage equipment with the most potential, and fuel cells are considered to be superexcellent clean energy (He and Chen, 2015; Peng et al., 2016; Yang et al., 2019). Oxygen reduction reaction (ORR) is the key to the performance of fuel cells (Yang et al., 2018; Zhao et al., 2018, 2019). So, it is very important to exploit suitable materials for a high performance supercapacitors and electrocatalysts for ORR. Among all materials, porous carbon is an excellent candidate material due to its good conductivity, large specific surface area, more active sites, hierarchical pore structure, and high chemical stability (Pan et al., 2014; Ma C. et al., 2017; Li et al., 2018; Liu B. et al., 2018).

Porous carbon is a kind of material with certain interconnected or closed pores. It is divided into microporous carbon, mesoporous carbon, macroporous carbon and hierarchical porous carbon (Liu et al., 2010). In recent decades, researchers have prepared many porous carbons with different structures and holes, including porous carbon nanofibers (Zhao et al., 2013; He et al., 2018), carbon nanotubes (Kshetri et al., 2018), graphene (Chao et al., 2018), metal-organic framework (MOF) derived porous carbon (Liu et al., 2017; Xia et al., 2017; Díaz-Duran and Roncaroli, 2018), and other new porous carbon materials based on different methods (He et al., 2019). Although these porous carbons have excellent performance, considering the complicated preparation processes and the high-cost, it is very difficult to prepare these porous carbons industrially (Chen et al., 2017). Therefore, it is highly necessary to exploit a simple and low-cost method to prepare porous carbon. Many researchers are very concerned about biomass carbon because it can be obtained from low-cost and renewable resources (Qian et al., 2015; Gao et al., 2017; Lu et al., 2017; Zhang et al., 2018; Zou et al., 2018; Li et al., 2019). Direct carbonization of biomass is an effective way to prepare porous carbon because the biomass owns various structures and pore distributions. Some hierarchical porous carbons were successfully synthesized from some biomass for a supercapacitor or electrocatalysts, such as 3D layer-stacking hierarchical porous carbon prepared from biomass tremella as electrode materials for a supercapacitor (Chen et al., 2018), porous carbon derived from coffee waste as an electrocatalyst (Chung et al., 2017), N-doped porous graphitic carbon nanosheets derived from Lycium barbarum L (Zuo et al., 2017), and N-doped porous carbon derived from water hyacinth (Liu et al., 2015) as electrocatalysts for oxygen reduction reaction (ORR), and 3D N-doped carbon nanofibers derived from bacterial cellulose as electrode materials for a supercapacitor (Chen et al., 2014). Although these porous carbons have achieved good performance, their structure and application are relatively simple. Hence, a new kind of biomass should be found to prepare porous carbon to realize versatile applications.

Juncus, a typical biomass feedstock, is a perennial herb aquatic plant. It can be widely found in warm regions of the world. In this work, N-doped hierarchical porous carbon (NDPC) derived from juncus with ZnCl_2_ as the activation reagent was prepared. NDPC were applied for a supercapacitor and ORR, respectively. The NDPC-3-800 was prepared by mixing juncus with ZnCl_2_ at a mass ratio of 1:3 and then carbonized at 800°C, it was used as electrode material of a supercapacitor and exhibited excellent specific capacitance of 290.5 F g^−1^ and 175.0 F g^−1^ at the current densities of 0.5 A g^−1^ and 50 A g^−1^, respectively. The supercapacitor showed good cycle stability and the capacitance was maintained at 94.5% after 10,000 cycles. The NDPC-5-800 exhibited outstanding ORR catalytic activity and stability.

## Experimental

### Materials Synthesis

Juncus was obtained from the local Changsheng Pharmacy (Nanchang, China). The juncus was washed with ethanol and deionized water, and then dried at 60°C. Subsequently, the pretreated juncus was directly carbonized at different temperatures in a nitrogen atmosphere for 120 min. After the reaction was cooled to room temperature naturally, the sample was collected and named hierarchical porous carbon (HPC-y), where y was the carbonization temperature.

**Scheme 1:**
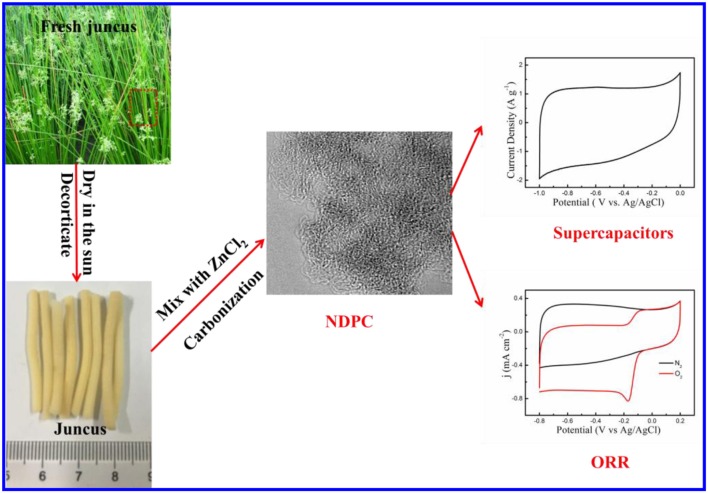
Schematic of the synthesis process of NDPC and its applications.

Furthermore, the pretreated juncus was mixed with ZnCl_2_ (the mass ratio was 1:1, 1:3 and 1:5, respectively) solution. After the evaporation of water, the juncus were carbonized in a ceramic crucible under a nitrogen atmosphere up to 800°C at a heating rate of 5°C min^−1^ then held for 120 min. After the reaction was cooled to room temperature naturally, the obtained materials were immersed in 2 M HCl solution for 24 h and washed repeatedly with deionized water until pH = 7 to remove the metal residue. Finally, it was dried in a vacuum at 60°C. The collected samples were named nitrogen doped porous carbon (NDPC-x-800), where x was the mass ratio of ZnCl_2_ to juncus. The preparation process is illustrated in Scheme 1.

### Material Characterization

Thermogravimetric analysis (TGA) was performed under a nitrogen flow rate of 50 ml min^−1^ at a heating rate of 10°C min^−1^ from room temperature to 900°C (HTC-3 Analyzer). Fourier transform infrared spectroscopy (FTIR) was recorded on Avatar 360 FTIR spectrometer (Nicolet). Brunauer-Emmett-Teller (BET) surface area and pore-size distribution were carried out by nitrogen adsorption and desorption (ASAP 2020 instrument). The surface morphology of the sample was observed by the HITACHI S-3400 N scanning electron microscope (SEM) and by JEM-2010 high resolution transmission electron microscopy (HRTEM). Elements presented in the samples were analyzed by energy dispersive X-ray spectroscopy (EDX). X-ray photoelectron spectroscopy (XPS) analysis was performed using an AXIS ULTRA DLD at an accelerating voltage of 15 kV. Powder X-ray diffraction (XRD) analysis was carried out on a D/Max 2500 V/PC X-ray powder diffractometer using Cu Kα radiation. Raman spectra were performed with a Lab RAM HR spectrometer at room temperature and an argon ion laser operating at a wave length of 632.8 nm as the excitation (Jobin Yvon Ltd).

### Electrochemical Characterization

Capacitance measurements were tested in a three or two electrodes system *via* a CHI760E (Chenhua, Shanghai, China) electrochemistry workstation. The working electrode was prepared by mixing the active material and carbon black with polytetrafluoroethylene (PTFE, used as the binder) in a weight ratio of 80:10:10. The mixture was then pasted on the nickel foam and dried at 80°C overnight. The mass loading of the working electrode was about 2.0 mg cm^−2^. The platinum wire and Ag/AgCl electrode was used as the counter electrode and reference electrode, respectively. In the two-electrode system, the above as-prepared working electrodes were used as positive and negative electrodes. All measurements were carried out in 6 M KOH solution.

The ORR electrochemical experiments were tested at room temperature with a three-electrode system. A platinum wire and an Ag/AgCl electrode (saturated KCl) were used as the counter and reference electrode, respectively. To prepare the working electrode, 5 mg active material was ultrasonically dispersed in 1 ml ultrapure water with 5% Nafion solution. Subsequently, 5 and 10 μl of the well-distributed catalyst ink were dropped onto the glassy carbon electrode (GCE) or rotation disk glassy carbon electrode (RD-GCE) surface, respectively, then dried at room temperature for electrochemical measurements. For comparison, the commercial Pt/C electrode was prepared in the same way. Both CV and ORR experiments were performed in 0.1 M KOH solution. Flowing N_2_/O_2_ and O_2_ was introduced into the alkaline solution to achieve N_2_/O_2_ and O_2_ saturated electrolyte solution for ORR and CV tests, respectively. ORR performance was conducted at different rotation rates from 400 to 2500 rpm with a scan rate of 10 mV s^−1^.

## Results and Discussion

**Figure 1:**
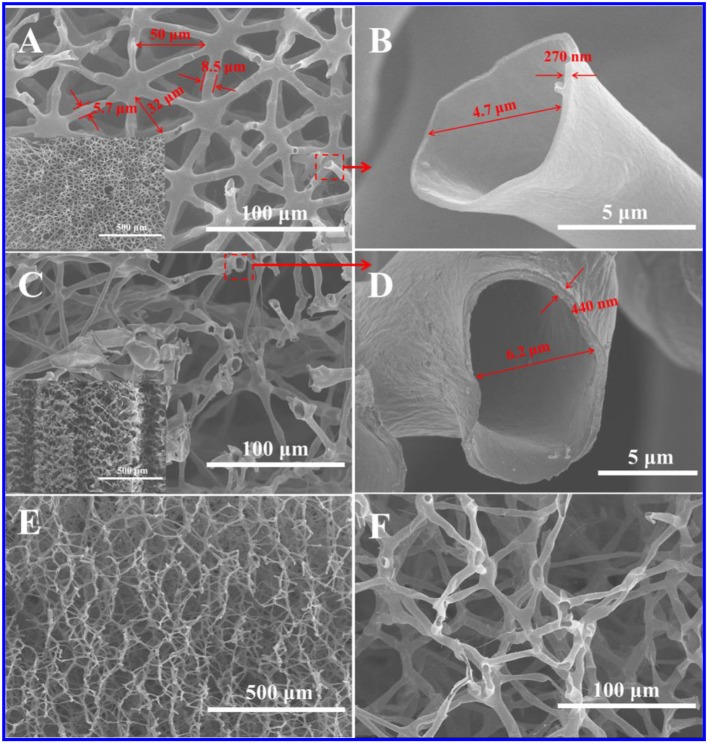
SEM images of HPC-800. **(A)** Top-view image (the inset was low magnification). **(B)** Image magnified from position A. **(C)** Side-view image (the inset was low magnification). **(D)** Image magnified from position C. **(E,F)** Longitudinal sectioned image.

Figure S1 showed that the weight loss of juncus was almost up to the maximum at 350°C, and about 30% of the mass was retained at 900°C. After the carbonation, the juncus slightly shrank while its shape was well kept (Figure S2). These carbonized products had a certain mechanical strength and could resist certain pressure. The top-view SEM image of juncus showed it had well-defined triangular networks with a size of 40–80 μm (Figure S3). These triangular networks were composited of tubular fibers with a diameter of about 10 μm and a wall thickness of about 1 μm. The side-view SEM image indicated that these triangular networks were cross-linked to each other in the longitudinal direction. After the carbonation, as shown in Figures 1A–F, the structure of HPC-800 was basically unchanged, but the size of the triangular network shrunk to 30–50 μm. The diameter of tubular fiber was decreased to 5–8 μm and the wall thickness of the tubular fiber was down to about 400 nm. The existence of C=O, C-O and C=N groups were confirmed by FTIR (Figures S4, S5).

**Figure 2:**
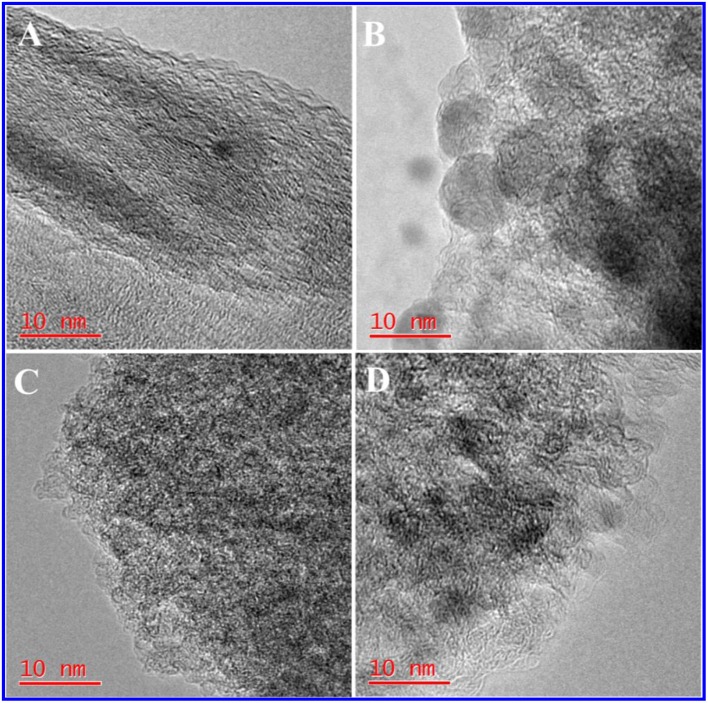
HR-TEM images of **(A)** HPC-800, **(B)** NDPC-1-800, **(C)** NDPC-3-800 and **(D)** NDPC-5-800.

**Figure 3:**
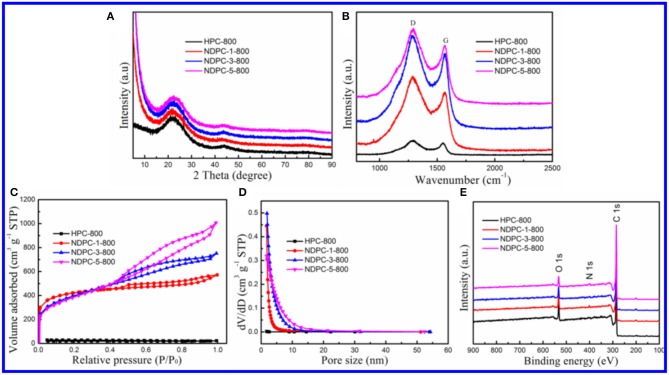
**(A)** XRD patterns of HPC-800, NDPC-1-800, NDPC-3-800 and NDPC-5-800. **(B)** Raman spectra of HPC-800, NDPC-1-800, NDPC-3-800 and NDPC-5-800. **(C)** Nitrogen adsorption-desorption isotherm and **(D)** pore size distributions of HPC-800, NDPC-1-800, NDPC-3-800 and NDPC-5-800. **(E)** The full-scan XPS spectra of HPC-800, NDPC-1-800, NDPC-3-800 and NDPC-5-800.

The TEM image of HPC-800 only showed a few micropores and mesopores (Figure 2A). After the activation of ZnCl_2_, many micropores and mesopores appeared in NDPC-1-800, NDPC-3-800 and NDPC-5-800 (Figures 2B–D). With the increase of ZnCl_2_, the carbon wall thickness of NDPC-x-800 decreased inch by inch and the number of pores in the NDPC-x-800 increased significantly (Gao et al., 2017) (Figure S6). The ZnCl_2_ is an activator which can effectively fabricate a large number of micropores as well as mesopores in the carbonization, which efficiently enlarged the surface area. Figure 3A shows the XRD characterization results of the samples. Two characteristic diffraction peaks appeared at 2θ =22.6° and 43°, belonging to the (002) disordered carbon and the (101) graphite carbon (Ding et al., 2015), respectively. Raman spectra showed two peaks (Figure 3B). The D peak belonged to disordered carbon and amorphous carbon around 1,320 cm^−1^, while the G peak belonged to an ordered graphite structure around 1,580 cm^−1^ (Naveen et al., 2017). The degree of graphitization was characterized by the ratio of peak strength of the D peak to the G peak (I_D_/I_G_). The I_D_/I_G_ increased from 1.02 for HPC-800 to 1.23 for NDPC-3-800. The results indicated that the graphitized structure was gradually damaged with the increased ZnCl_2_, and in the activation process, ZnCl_2_ as an activator destroyed the carbon structure, leading to the increase of an irregular carbon structure.

Figures 3C,D showed the BET specific surface areas and pore size distribution of HPC-800 and NDPC-x-800, respectively. The nitrogen adsorption and desorption curve of NDPC-x-800 did not coincide exactly. The hysteresis loop could be observed within the interval of the relative pressure (P/P_0_) 0.45 to 0.95, indicating the existence of partial micropores and mesopores in the samples (Gao et al., 2015). The results of the specific surface area, pore volume, and pore size are shown in Table 1. A high specific surface area facilitated the storage of more electrolyte ions and formed a double layer capacitance (Gupta et al., 2015). The strong electric potential in the micropores of activated carbon could enhance the double layer capacitance and the mesopores could reduce the resistance of electrolyte ions in the electrode materials. Therefore, the high specific surface area and porous structure of NDPC-3-800 could achieve higher specific capacitance. Others such as the high specific surface area and high pore volume of NDPC-5-800 were conducive to provide more catalytic active sites, facilitating good contact between the active site and oxygen molecules, then promoting the oxygen reduction performance.

**Table 1:** Specific surface area, pore structure characterization parameters and elemental analysis of different samples.

**Samples**	**Specific surface area (m^**2**^ g^**−1**^)**	**Total pore volume (m^**3**^ g^**−1**^)**	**Average pore diameter (nm)**	**Elemental analysis**		
				**C%**	**N%**	**O%**
HPC-800	57.8	0.035	9.1	91.7	1.0	7.3
NDPC-1-800	1344.7	0.885	3.2	90.4	1.8	7.8
NDPC-3-800	1379.9	1.163	3.6	90.7	1.8	7.5
NDPC-5-800	1367.2	1.559	4.5	91.3	2.1	6.6

The full XPS spectrum showed that HPC-800, NDPC-1-800, NDPC-3-800, and NDPC-5-800 were mainly composed of C, N, and O elements (Figure 3E). There were three peaks at about 284.8 eV, 400.9 eV, and 533.2 eV corresponding to C 1s, N 1s, and O 1s, respectively (Yuan et al., 2015). Figure S9A shows that the high-resolution C 1s XPS spectrum of NDPC-3-800 could be fitted into three peaks at 284.6 eV, 285.4 eV, and 288.7 eV, corresponding to C-C=C, C-N, and C=O, respectively. Figure S9B shows that the high-resolution N 1s XPS spectrum of NDPC-3-800 could be fitted into four peaks at 397.3, 398.5 eV, 400.7 eV, and 403.9 eV, corresponding to pyridinic N, pyrrolic N, graphitic N, and oxidic N, respectively (Lin et al., 2015). Figure S9C shows that the high-resolution O 1s XPS spectrum of NDPC-3-800 could be fitted into three peaks at 531.4 eV, 533.3 eV, and 535.9 eV, corresponding to C=O, C-OH, and C-O-C, respectively. These doped nitrogen elements could greatly enhance the performance of electrochemical supercapacitors and ORR (Yang et al., 2015). Furthermore, graphitic N could also promote electron transport and improve the performance of electrochemical energy storage. The high-resolution XPS spectra of HPC-800, NDPC-1-800, and NDPC-5-800 are shown in Figures S7, S8, S10, respectively.

**Figure 4:**
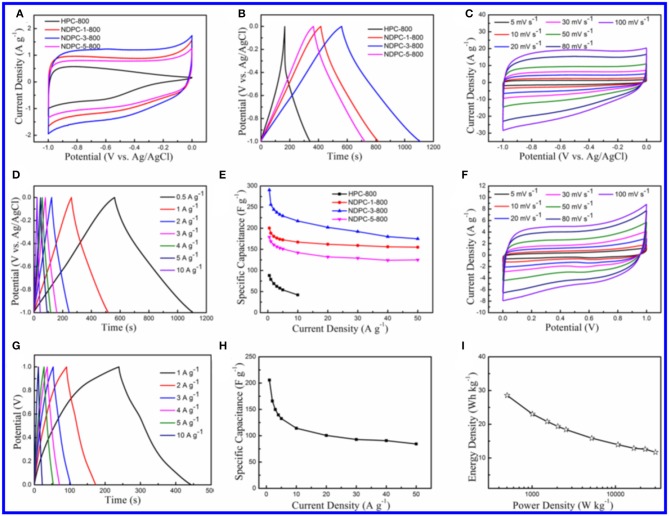
**(A)** CV curves of HPC-800, NDPC-1-800, NDPC-3-800, and NDPC-5-800 electrodes in 6.0 M KOH electrolyte at the scan rate of 5 mV s^−1^. **(B)** Galvanostatic charge-discharge curves of HPC-800, NDPC-1-800, NDPC-3-800 and NDPC-5-800 electrode at the current density of 0.5 A g^−1^. **(C)** CV curves of NDPC-3-800 electrode at different scan rate. **(D)** Galvanostatic charge-discharge curves of NDPC-3-800 electrode at different current density. **(E)** Specific capacitance of HPC-800, NDPC-1-800, NDPC-3-800 and NDPC-5-800 electrode at various current densities. **(F–I)** Electrochemical characterization of NDPC-3-800//NDPC-3-800 symmetric supercapacitor device in 6.0 M KOH electrolyte. **(F)** CV curves at different scan rates. **(G)** Galvanostatic charge-discharge curves at different current density. **(H)** Specific capacitances of the device at various current densities. **(I)** Ragone plot.

The electrochemical performance of the as-prepared materials was tested and is shown in Figure 4. Figure 4A shows the CV curves of the as-prepared materials between potential windows of−1.0 to 0.0 V. Obviously, CV curves of HPC-800 exhibited a small irregular shape. Inversely, CV curves of NDPC-1-800, NDPC-3-800, and NDPC-5-800 showed rectangular-like shapes with a minor redox hump, indicating an ideal double layer capacitive which is due to the higher specific surface area and the N-doped contribution of the pseudocapacitance. Figure 4A shows that NDPC-3-800 had the largest CV area, indicating the largest specific capacitance. Figure 4B shows the galvanostatic charge-discharge (GCD) curves of the materials at 0.5 A g^−1^, exhibiting a symmetrical triangular shape. The specific capacitance of NDPC-3-800 was up to 290.5 F g^−1^ which was higher than that of HPC-800 (88.2 F g^−1^), NDPC-1-800 (200.1 F g^−1^), and NDPC-5-800 (179.0 F g^−1^). Figure 4C and Figure S11 show the CV curves of HPC-800, NDPC-1-800, NDPC-3-800, and NDPC-5-800 electrodes at different scan rates. All curves displayed rectangular-like shapes except that of the HPC-800 electrode (Figure S11A). As the scan rate increased, the shape of the CV curve remained good. The result showed that electrode processes exhibited a fast response. Figure 4D and Figure S12 show the GCD curves of different electrodes at various current densities under the potential window of −1.0 to 0.0 V. The good symmetry of charging-discharging curves suggested that the electrode had good electrochemical reversibility. Figure 4E shows the comparison of specific capacitance of different electrodes at different current densities. For the NDPC-3-800 electrode, when the current density was increased to 10 A g^−1^, the specific capacitance remained at the original 74.7% (217.0 F g^−1^). This result was higher than that of HPC-800 (47.6%). When the current density was increased to 50 A g^−1^, the specific capacitance of NDPC-3-800 was still as high as 175.0 F g^−1^, higher than that of both NDPC-1-800 (155.0 F g^−1^) and NDPC-5-800 (125.0 F g^−1^). In addition, the NDPC-3-800 electrode showed higher specific capacitance than many previously reported biomass materials derived carbon electrode (Table S1). The excellent electrochemical performance of NDPC-3-800 might be due to its large specific surface area, hierarchical pores, high pore volume and effective nitrogen doping, which provided fast and efficient charge transfer and good electrolyte penetration. As shown in Figure S18, the charge transfer resistance of all these electrodes was very small. Compared with HPC-800, NDPC-1-800, and NDPC-5-800, NDPC-3-800 possessed lower equivalent series resistances in the expanded high frequency region (inset of Figure S13) and a more vertical line in low frequency.

In order to explore the practical application of the NDPC-3-800 electrode in the field of energy storage, a symmetric supercapacitor was prepared using NDPC-3-800 as both positive and negative electrodes. The NDPC-3-800//NDPC-3-800 symmetric supercapacitor showed a rectangular shaped CV curve which is the characteristic of an ideal electrochemical double-layer capacitor (Figure 4F). With the increase of scan rate, the shape of the CV curve remained rectangular, indicating a rapid current-potential response. Figure 4G shows the GCD curves at different current densities from 1 to 10 A g^−1^. All GCD curves were approximately symmetrical and linear, indicating excellent electrochemical reversibility and ideal double-layer capacitance. Figure 4H shows the specific capacitance at different current densities. The NDPC-3-800//NDPC-3-800 symmetric supercapacitor showed the specific capacitance of 205.7 F g^−1^ at the current density of 1 A g^−1^ and the specific capacitance of 84.4 F g^−1^ at 50 A g^−1^. The nearly vertical Nyquist plot showed a good electrical double-layer capacitor (Figure S14). The energy density and power density of the NDPC-3-800//NDPC-3-800 symmetric supercapacitor was 28.6 Wh kg^−1^ at 502.5 W kg^−1^ and 11.7 Wh kg^−1^ even when the power density was elevated to 30153.2 W kg^−1^ (Figure 4I). The capacities of the symmetric supercapacitor remained 94.5% initial capacitance over 10,000 cycles at the current density of 10 A g^−1^, indicating a good cycling stability (Figure S15).

**Figure 5:**
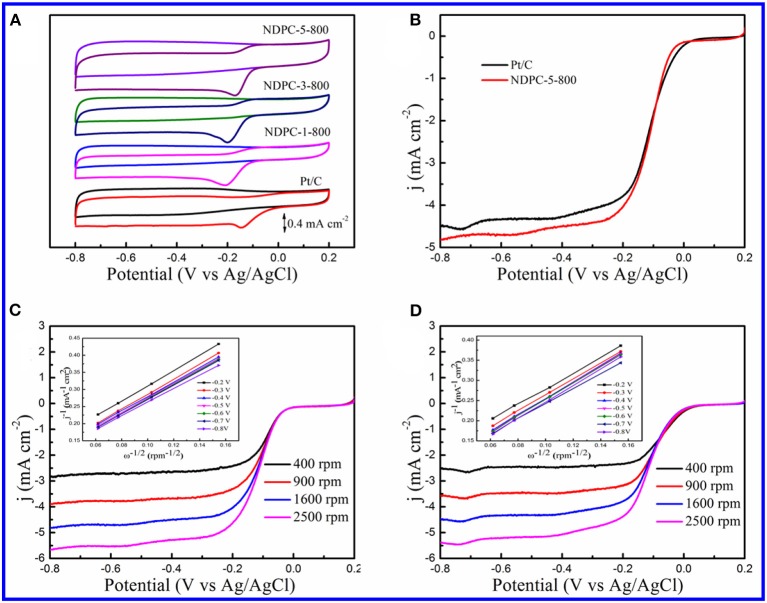
**(A)** CV curves of Pt/C, NDPC-1-800, NDPC-3-800 and NDPC-5-800 in N_2_ and O_2_ saturated 0.1 M KOH at the scan rate of 10 mV s^−1^. **(B)** LSV curves of NDPC-5-800 and Pt/C catalyst recorded in O_2_ saturated 0.1 M KOH at the scan rate of 10 mV s^−1^ and the rotation rate of 1600 rpm. **(C)** RDE voltammograms of NDPC-5-800 catalyst in O_2_ saturated 0.1 M KOH with various rotation rates at scan rate of 10 mV s^−1^. Inset showed the corresponding Koutecky–Levich plots of NDPC-5-800 catalyst at different potentials. **(D)** RDE voltammograms of Pt/C catalyst in O_2_ saturated 0.1 M KOH with various rotation rates at the scan rate of 10 mV s^−1^. Inset showed the corresponding Koutecky–Levich plots of Pt/C catalyst at different potentials.

In order to study the electrocatalytic activity of the materials for ORR, CV curves were recorded in a potential window from −0.8 to 0.2 V at 10 mV s^−1^ in N_2_ or O_2_ saturated 0.1 M KOH electrolyte (Figure 5A, Figure S16). In N_2_ saturated solution, no obvious peaks were observed in the CV curves for all the catalysts, while the CV curves showed a reduction peak of oxygen in the O_2_ saturated solution. The reduction peak was much more negative without the activation of ZnCl_2_ (Figure S16). But as shown in Figure 5A, a significant reduction peak around −0.173 V was observed on the NDPC-5-800 catalyst in the O_2_ saturated electrolyte, which was very close to that of a Pt/C catalyst of about −0.145 V. In addition, the NDPC-5-800 catalyst showed good catalytic activity than many previously reported biomass materials derived carbon catalysts (Table S2). The LSV curves had also been recorded at a rotation rate of 1,600 rpm for Pt/C and NDPC-5-800 catalysts (Figure 5B). The NDPC-5-800 catalyst displayed the most positive onset potential (E_onset_) of 0 V which was very close to that of the Pt/C catalyst at 0.021 V, the most positive half-wave potential (E_1/2_) of −0.113 V which was very close to that of Pt/C catalyst at −0.103 V. However, NDPC-5-800 catalyst displayed higher kinetic-limiting current density of 4.51 mA cm^−2^ at −0.40 V, slightly higher than the Pt/C catalyst of 4.30 mA cm^−2^. Figures 5C,D showed a battery of LSV curves at different rotation rates for NDPC-5-800 and Pt/C catalysts. The corresponding K-L plots derived from the RDE voltammograms are presented in insets of Figures 5C,D. The K-L plots at various potentials exhibited good linearity for both NDPC-5-800 and Pt/C catalysts, which indicated the first-order reaction kinetics for the ORR with respect to the concentration of dissolved oxygen (Jiang et al., 2019). In addition, it also demonstrated a four-electron ORR pathway. For practical application, the toleration to methanol cross-over was tested in O_2_ saturated 0.1 M KOH solution at 50 mV s^−1^ (Figure S17). When methanol was added, the CV curves of the NDOC-5-800 catalyst had no significant change, indicating its good resistance to methanol crossover effect (Liu F. et al., 2018). However, for Pt/C catalyst, when methanol was added, the ORR peak disappeared, and a pair of new peaks appeared. A cycling stability test was carried out at 50 mV s^−1^ (Figure S18), and the CV curves of the NDOC-5-800 catalyst exhibited a negligible attenuation after 1,000 cycles. The above results indicated that the NDPC-5-800 catalyst had excellent ORR catalytic activity and stability in alkaline medium.

## Conclusion

In summary, novel carbon materials derived from biomass juncus was prepared through a simple, low-cost and environmentally friendly one-step method. NDPC-x-800 with a high specific surface area, more active sites, more pores, and ionic channels was prepared by carbonization of a mixture of crude juncus and ZnCl_2_. The as-prepared NDPC-3-800 was used as a supercapacitor electrode and exhibited high specific capacitance of 290.5 F g^−1^ at 0.5 A g^−1^ and good rate capability. In addition, the NDPC-3-800//NDPC-3-800 symmetric supercapacitor showed high energy density (28.6 Wh kg^−1^), high power density (30153.2 W kg^−1^), and good cycling stability after 10,000 cycles with 94.5% capacitance retention. The NDPC-5-800 exhibited excellent ORR catalytic activity and stability, attributed to their high surface area and more active sites. More importantly, it had resistance to methanol cross-over. Therefore, juncus as a renewable material can derive various materials for potential application in different fields, to achieve energy sustainable development and to remove pollutants from the environment.

## Data Availability Statement

The datasets generated for this study are available on request to the corresponding author.

## Author Contributions

GH and LW conceived the idea. GH, GP, YS, and LW designed and fabricated the sample, and conducted the experiment. All authors contributed to the analysis of data and the draft of the manuscript.

### Conflict of Interest

The authors declare that the research was conducted in the absence of any commercial or financial relationships that could be construed as a potential conflict of interest.
