# Supplementary material for: Biomass Juncus Derived Nitrogen-Doped Porous Carbon Materials for Supercapacitor and Oxygen Reduction Reaction
Source: Front Chem. 2020 Apr 15;8:226. doi: 10.3389/fchem.2020.00226 (PMC7174754; doi:10.3389/fchem.2020.00226)
Supplement: Supplementary file 1 [file Data_Sheet_1.pdf]

Supplementary Information

Biomass juncus derived nitrogen-doped porous carbon materials for supercapacitor and oxygen reduction reaction

Guanghua He[1,2*], Genping Yan[1]， Yonghai Song[2] and Li Wang[2*]

[1]Engineering & Technology Research Center for Environmental Protection Materials and Equipment of Jiangxi Province， College of materials and chemical engineering, Pingxiang University, Pingxiang, 337055, China

[2] Key Laboratory of Functional Small Organic Molecule, Ministry of Education, Key Laboratory of Chemical Biology, Jiangxi Province, College of Chemistry and Chemical Engineering, Jiangxi Normal University, Nanchang, 330022, China.

∗Corresponding author: Tel/Fax: +86 0791 88120861.

E-mail: guanghuaonly@163.com (G. He), lwanggroup@aliyun.com (L. Wang).

Electrochemical calculation

In the three-electrode system, the specific capacitance (C_m, F g^{-1}) of the electrode is calculated from the galvanostatic charge-discharge (GCD) curves based on the following equation:

$C_m=I\Delta t/(m\Delta V)$

where I (A) is the discharging current, Δt (s) is the discharging time, m (g) is the mass loading of active material in the working electrode and ΔV (V) is the discharging potential range.

In the two-electrode system, the specific capacitance (C_s, F g^{-1}) of the electrode is calculated from the GCD curves based on the following equation:

$C_s=2I\Delta t/(m\Delta V)$

where I (A) is the discharging current density, Δt (s) is the discharging time, m (g) is the mass loading of active material based on one electrode and ΔV (V) is the discharging potential range.

The energy density and power density are calculated by using the following equations:

$E=C_{sc}(\Delta V)^2/(2\times3.6)$

$P=3600E/\Delta t$

where E (Wh kg^{-1}) is the specific energy density, P (W kg^{-1}) is the specific power density of the symmetrical supercapacitor system, C_{sc} (F g^{-1}) is the gravimetric specific capacitance of the total symmetrical system, Δ V (V) is the operation voltage for charging and discharging, and Δt (s) is the discharging time. Electrochemical impedance spectroscopy (EIS) is obtained in a frequency range of 100 kHz to 10 mHz at open circuit voltage with an alternate current amplitude of 5 mV.

The kinetic current density (J_k) and the number of electrons transferred (n) are determined from detailed analysis based on the Koutecky-Levich (K-L) equation, at different electrode potentials:

$1/J=1/J_k+1/(B\omega^{0.5})$

$B = 0.62nFC_0(D_0)^{2/3}v^{-1/6}$

Where J is the measured current density, J_k is the kinetic current density, and ω is the electrode rotating rate. B can be determined from the slopes of K-L plots based on the K-L equation, where n represents the number of electrons transfer per O_2 molecule, F is the Faraday constant (F=96485 C mol^{-1}), C_0 denotes the bulk concentration of O_2 (1.26×10^{-3} mol L^{-1}), D_0 is the diffusion coefficient of O_2 (1.93×10^{-5} cm^2 s^{-1}), and υ is the kinetic viscosity of the electrolyte (1.0×10^{-2} cm^2 s^{-1}).

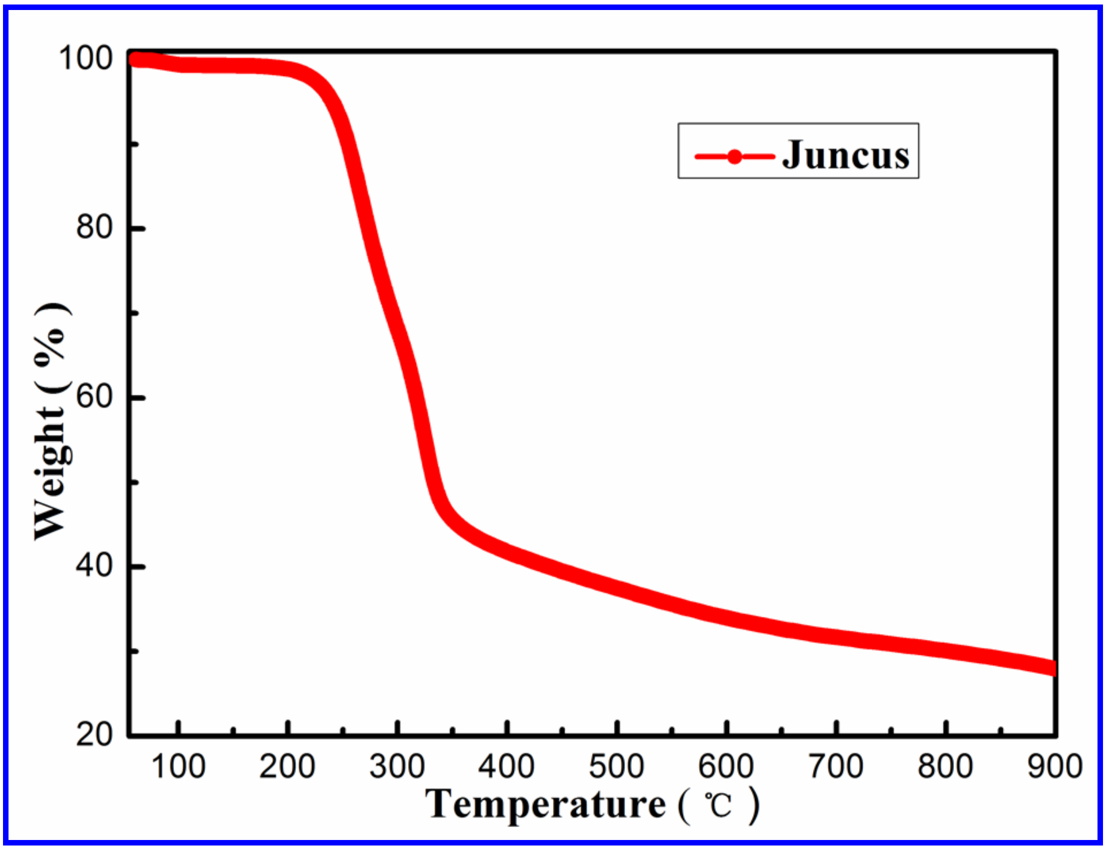

Figure S1. Thermal gravimetric analyses curve of juncus.

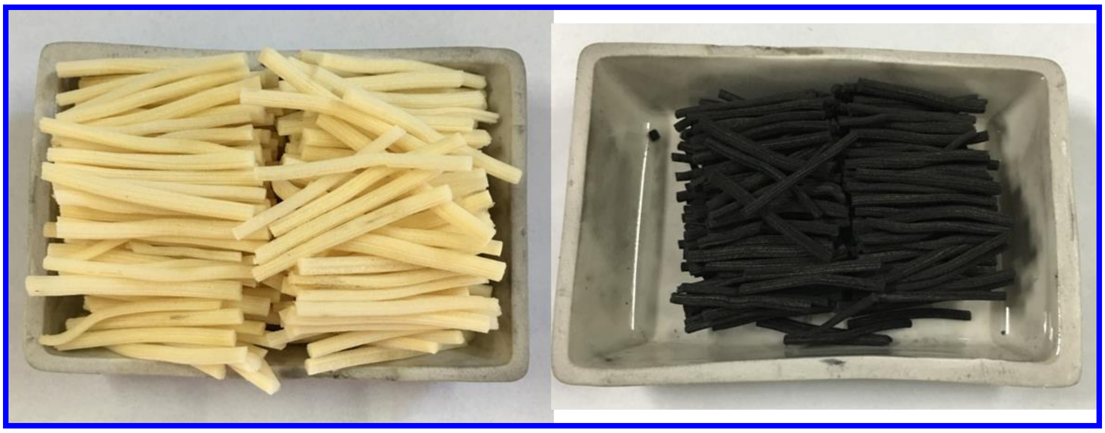

Figure S2. Photos of juncus and carbonated juncus (HPC-800).

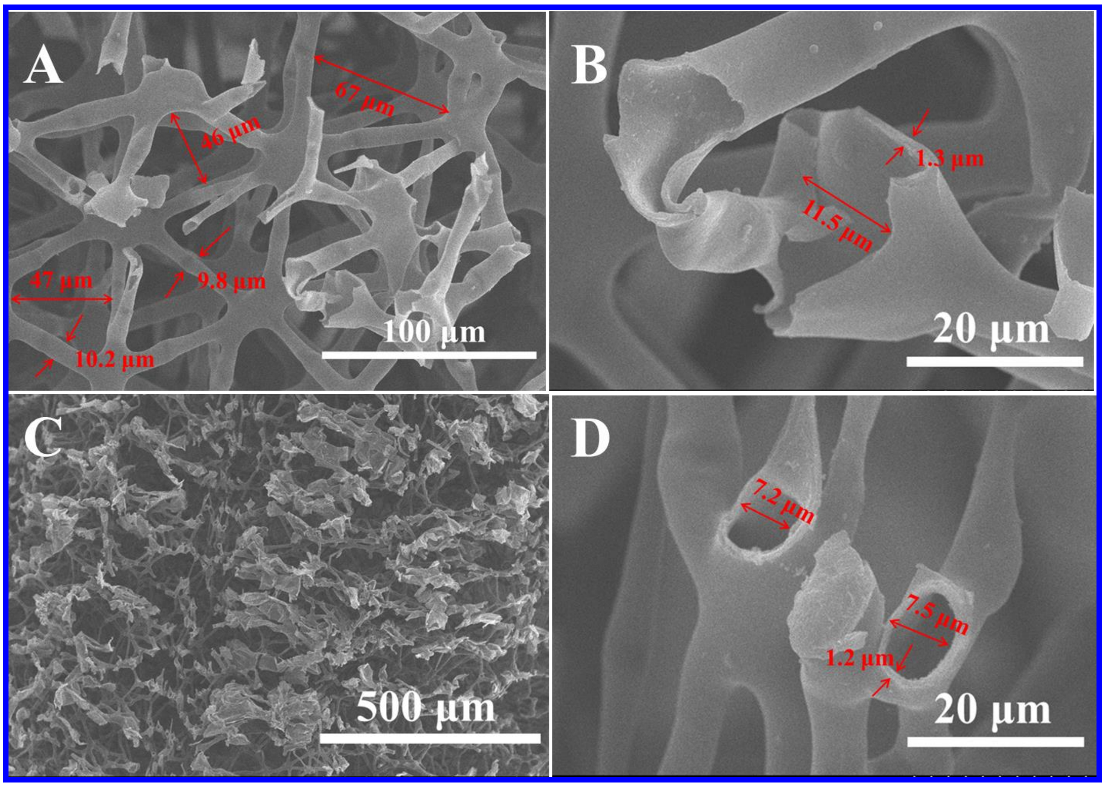

Figure S3. SEM images of crude juncus, (A) top-view image; (B) image magnified from position A; (C) side-view image; (D) image magnified from position C.

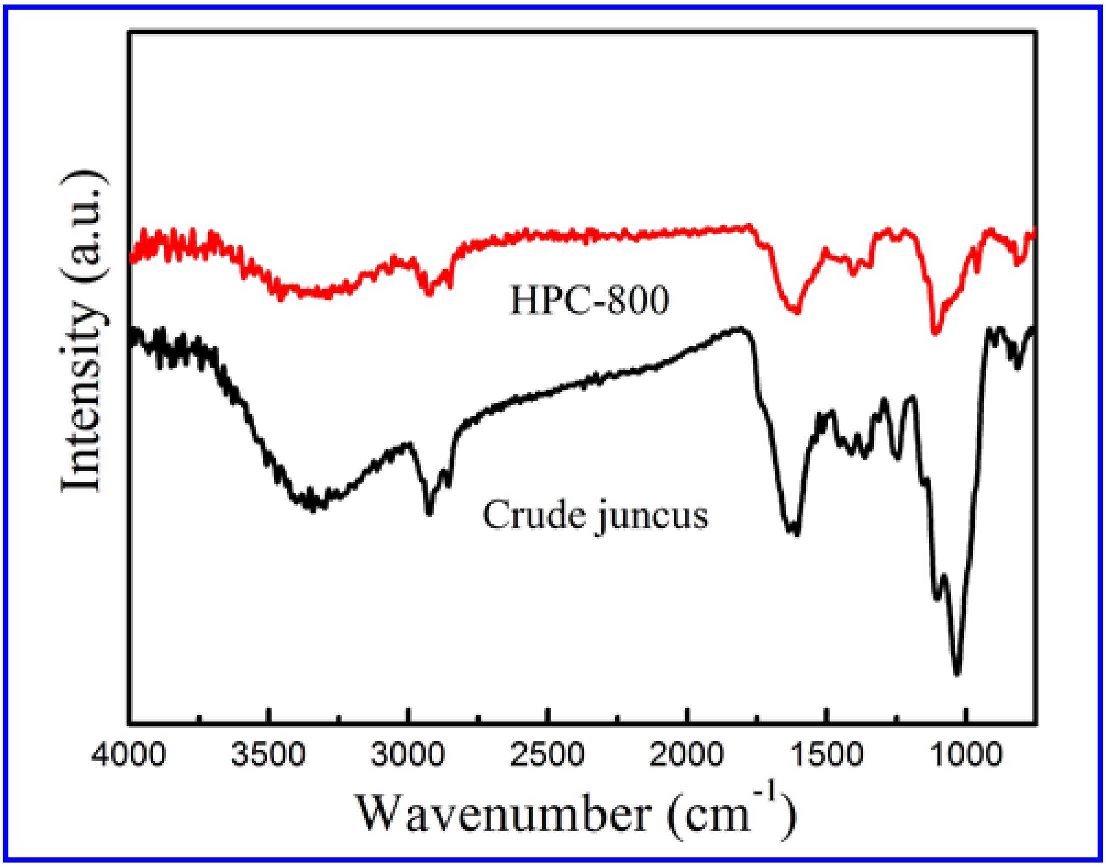

Figure S4. FT-IR spectra of crude juncus and HPC-800.

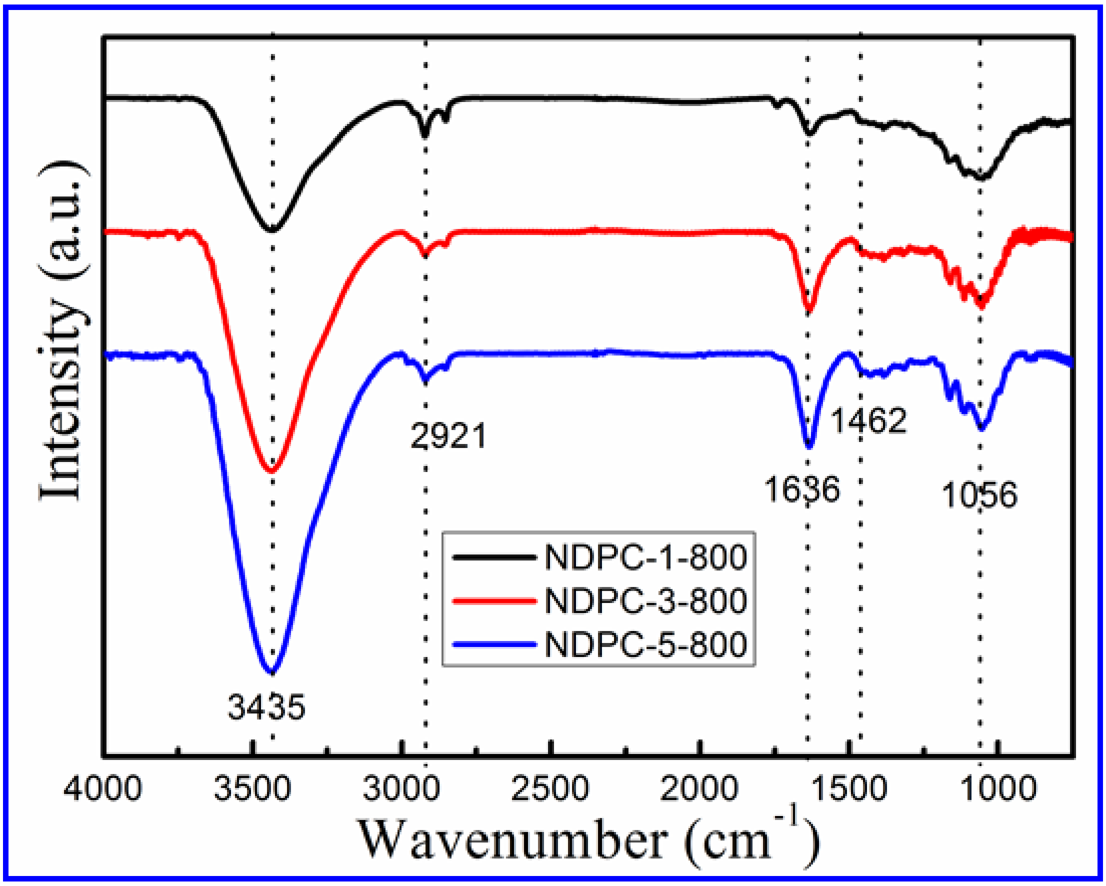

Figure S5. FT-IR spectra of NDPC-1-800, NDPC-3-800 and NDPC-5-800.

In order to explore the chemical functional groups of the samples, FTIR was collected and shown in Figure S4 and Figure S5. It can be seen from Figure S4 that several peaks representing oxygen-contained groups, such as C=O (1636 cm^{-1}, 2921 cm^{-1}), C-O (1062 cm^{-1}) and -OH (3435 cm^{-1}), were observed in the crude juncus. However, these peaks became weak or disappeared once the crude juncus were processed to HPC-800. The reason was that hydrophilic groups were removed during heat treatment. However, it can be observed from Figure S5 that the strong peak at 3435 cm^{-1} was assigned to -OH stretching vibration, the peak approach 2921 cm^{-1} and 1636 cm^{-1} derived from C=O stretching vibration, the peak around 1462 cm^{-1} should be attributed to C=C and C=N bands, and the broad peak centered at 1056 cm^{-1} was characteristic of C-O bonds. These results illustrated that O and N elements existed in NDPC-x-800 and gave these with high conductivity and good surface wettability, which was effective in improving electrochemical performance.

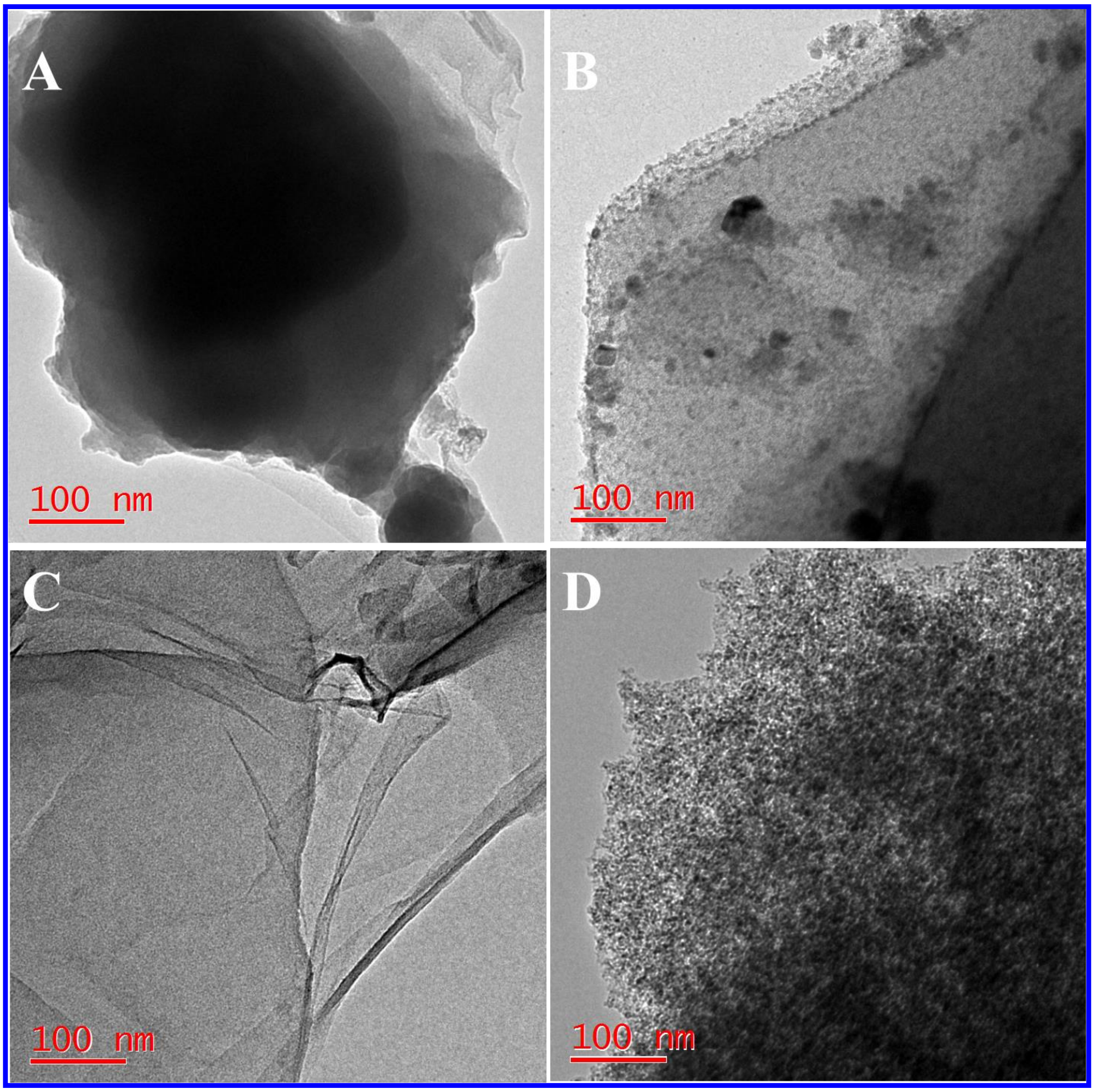

Figure S6. TEM of HPC-800 (A), NDPC-1-800 (B), NDPC-3-800 (C) and NDPC-5-800 (D).

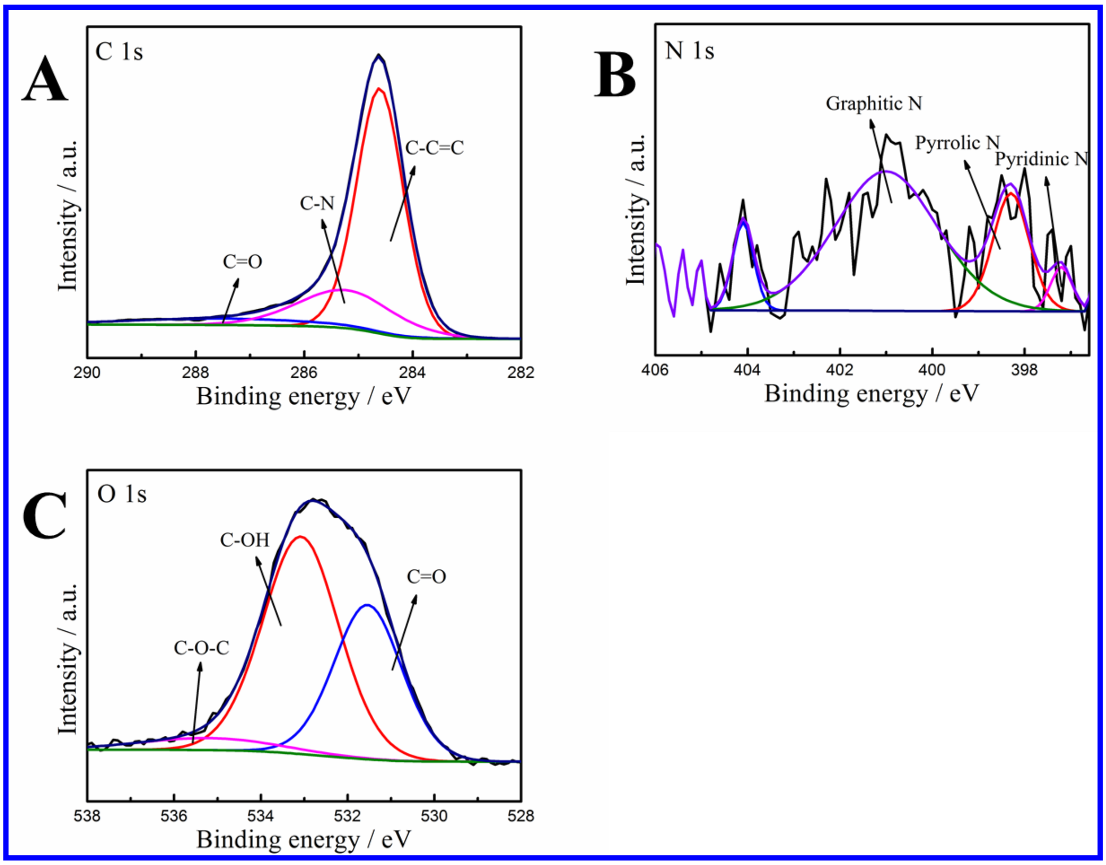

Figure S7. High-resolution XPS spectra of C1s (A), N1s (B) and O1s (C) of HPC-800.

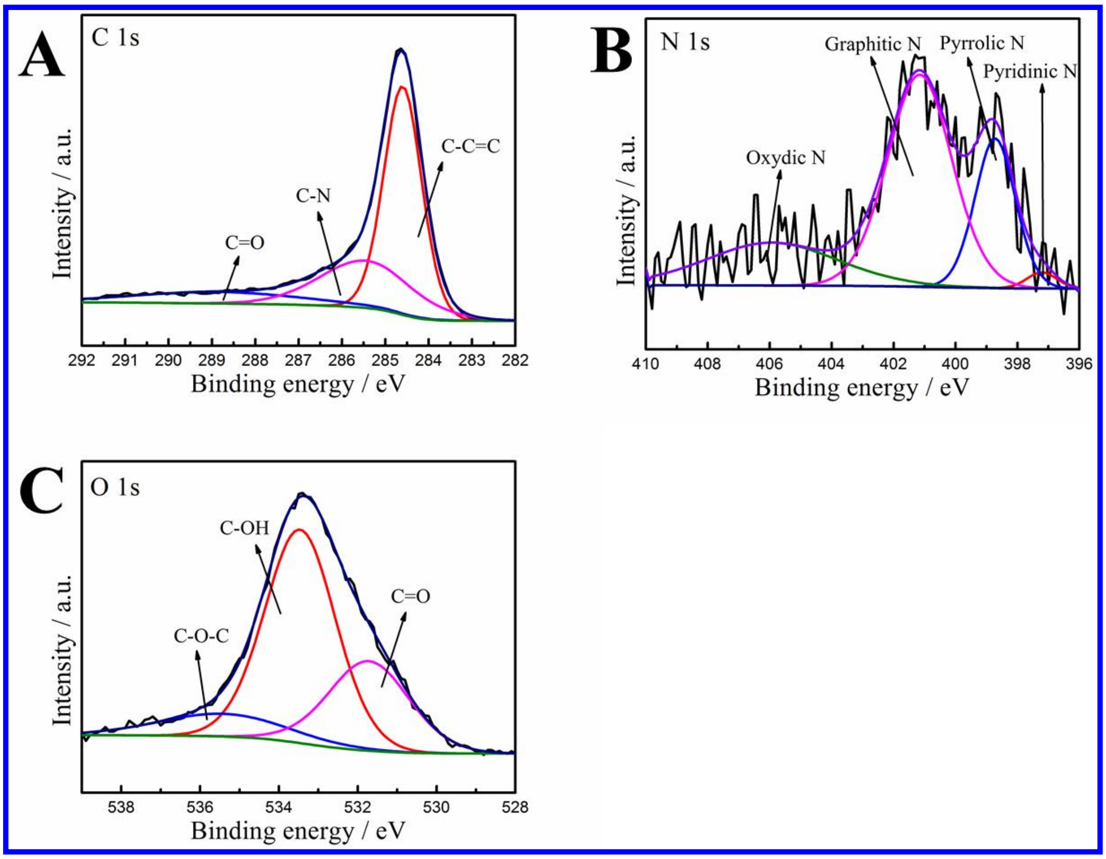

Figure S8. High-resolution XPS spectra of C1s (A), N1s (B) and O1s (C) of NDPC-1-800.

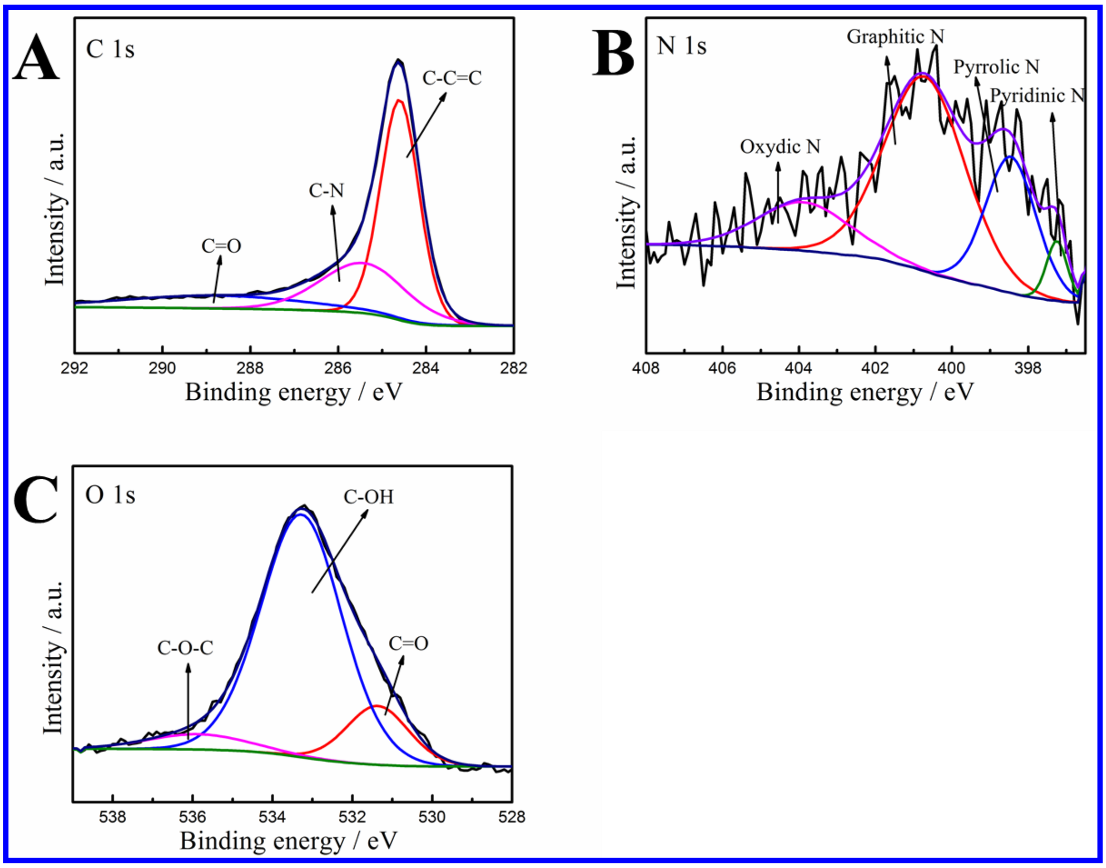

Figure S9. High-resolution XPS spectra of C1s (A), N1s (B) and O1s (C) of NDPC-3-800.

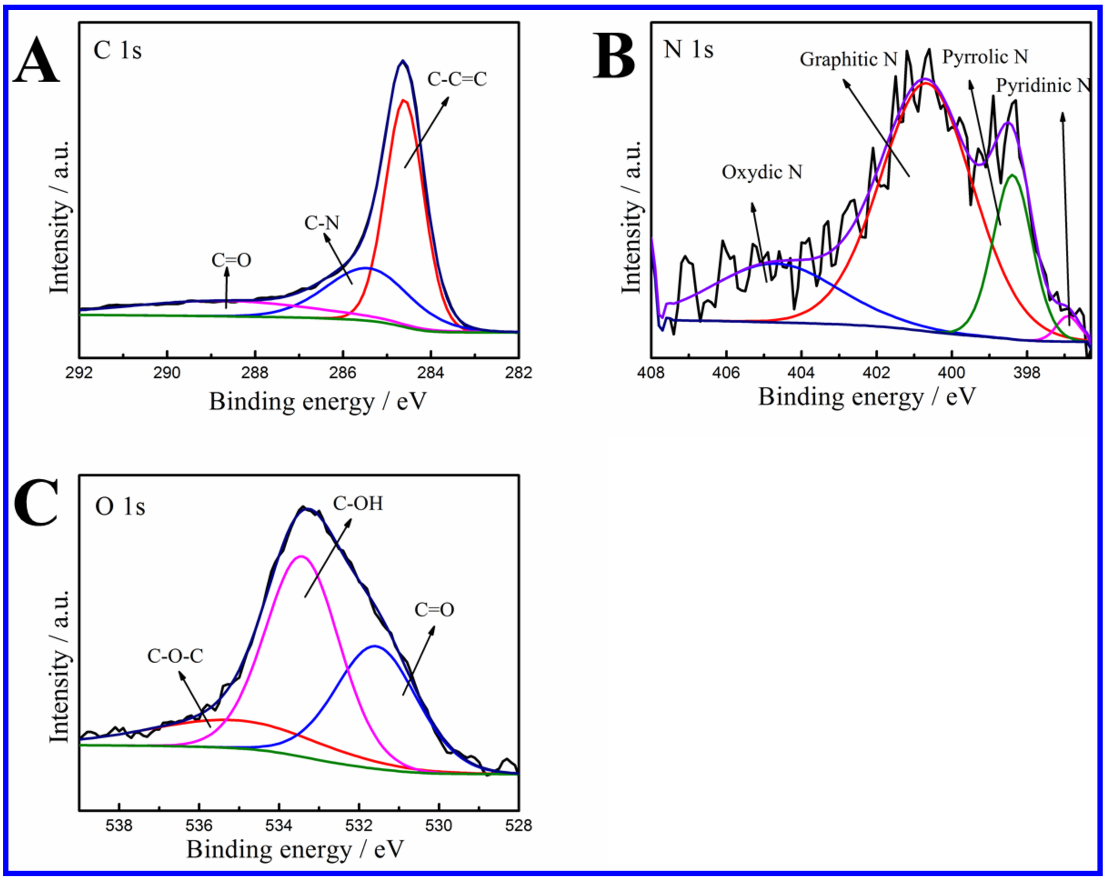

Figure S10. High-resolution XPS spectra of C1s (A), N1s (B) and O1s (C) of NDPC-5-800.

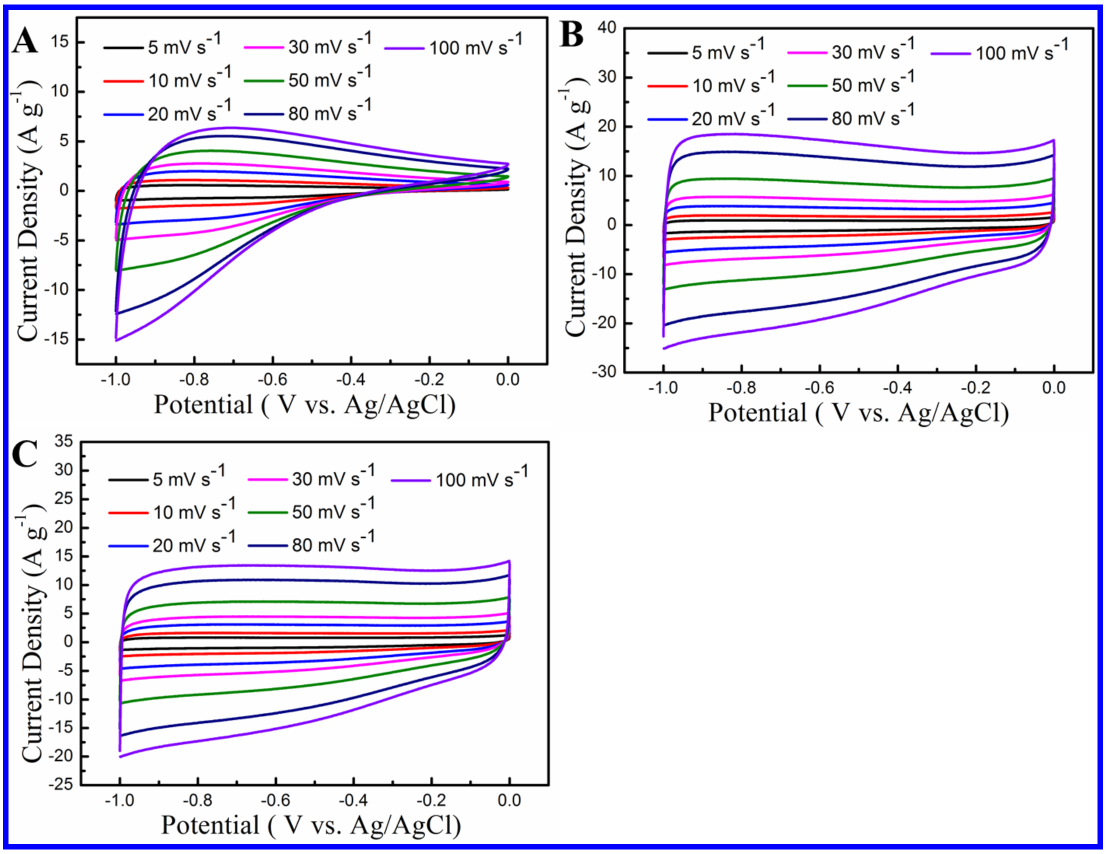

Figure S11. Cyclic voltammogram curves of (A) HPC-800; (B) NDPC-1-800and (C) NDPC-5-800 electrode at different scan rate.

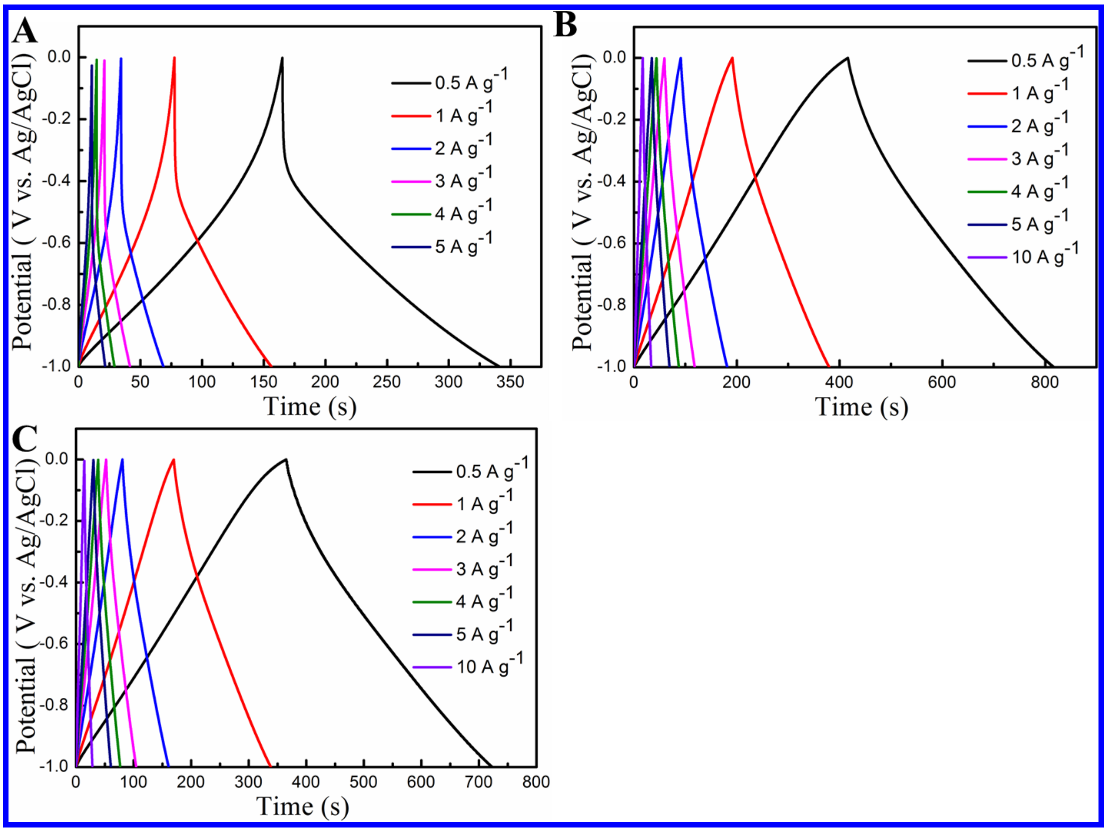

Figure S12. Galvanostatic charge-discharge curves of (A) HPC-800; (B) NDPC-1-800 and (C) NDPC-5-800 electrode at different current density.

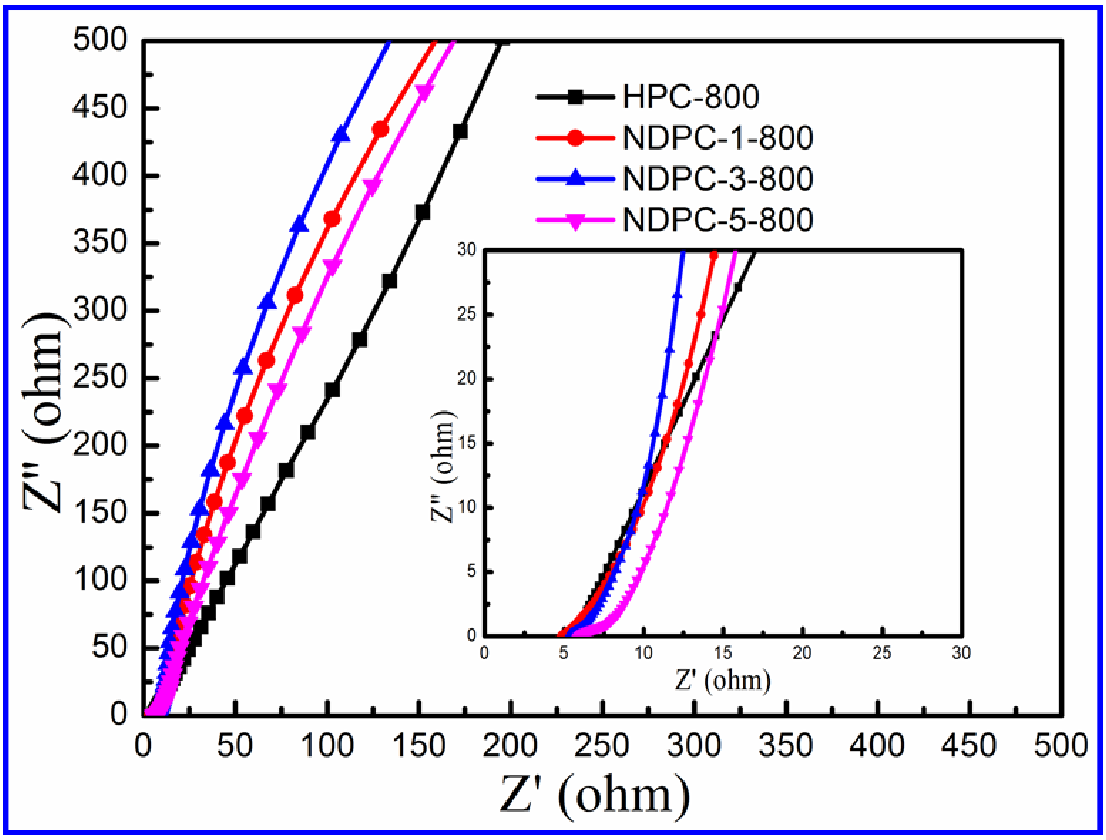

Figure S13. Nyquist plots of HPC-800, NDPC-1-800, NDPC-3-800 and NDPC-5-800 electrode.

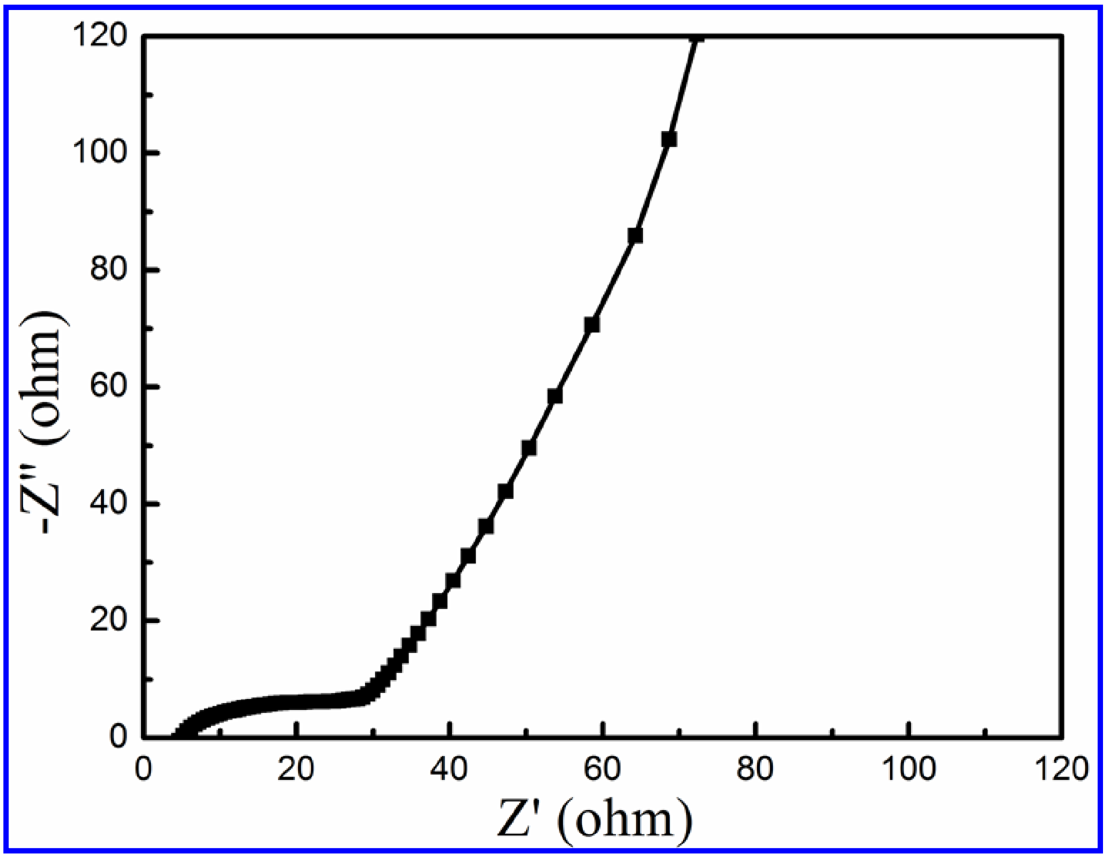

Figure S14. Nyquist plot of NDPC-3-800//NDPC-3-800 symmetric supercapacitor device in 6.0 M KOH electrolyte.

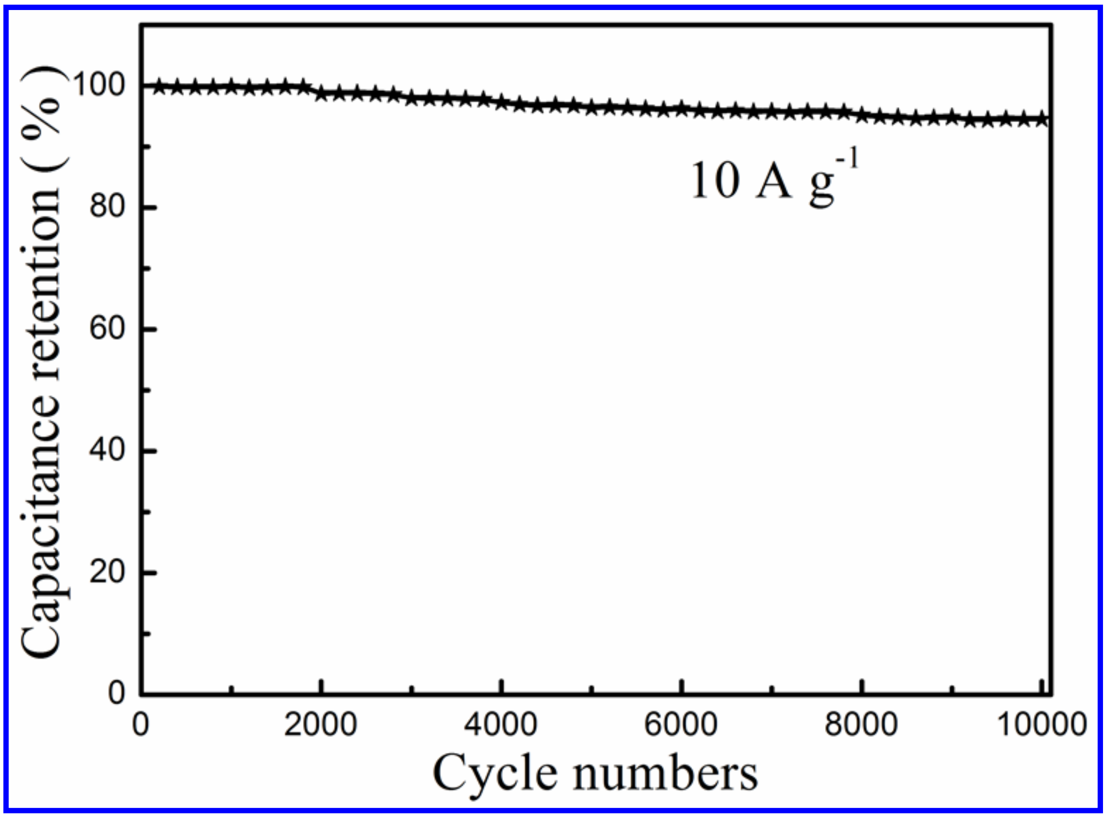

Figure S15. The cycling stability of NDPC-3-800//NDPC-3-800 symmetric supercapacitor device at the current density of 10 A g^{-1} in 6.0 M KOH electrolyte.

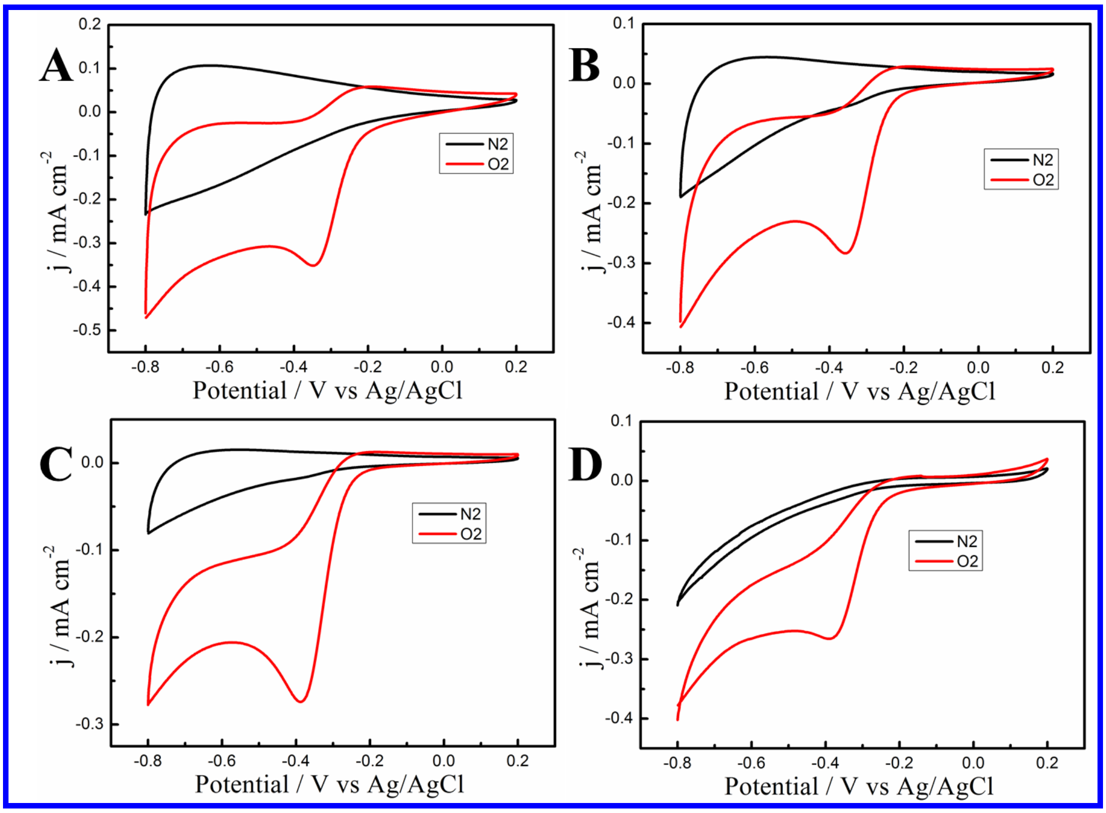

Figure S16. CV curves of HPC-700 (A), HPC-800 (B), HPC-900 (C) and HPC-1000 (D) in N_2 and O_2 saturated 0.1 M KOH at the scan rate of 10 mV s^{-1}.

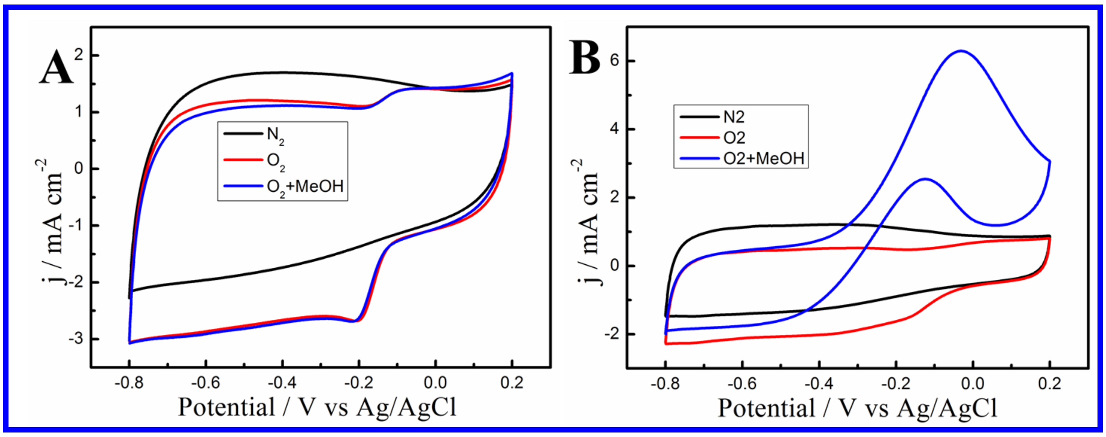

Figure S17. The resistance to methanol cross-over effect of (A) NDPC-5-800 and (B) Pt/C test at the scan rate of 50 mV s^{-1}.

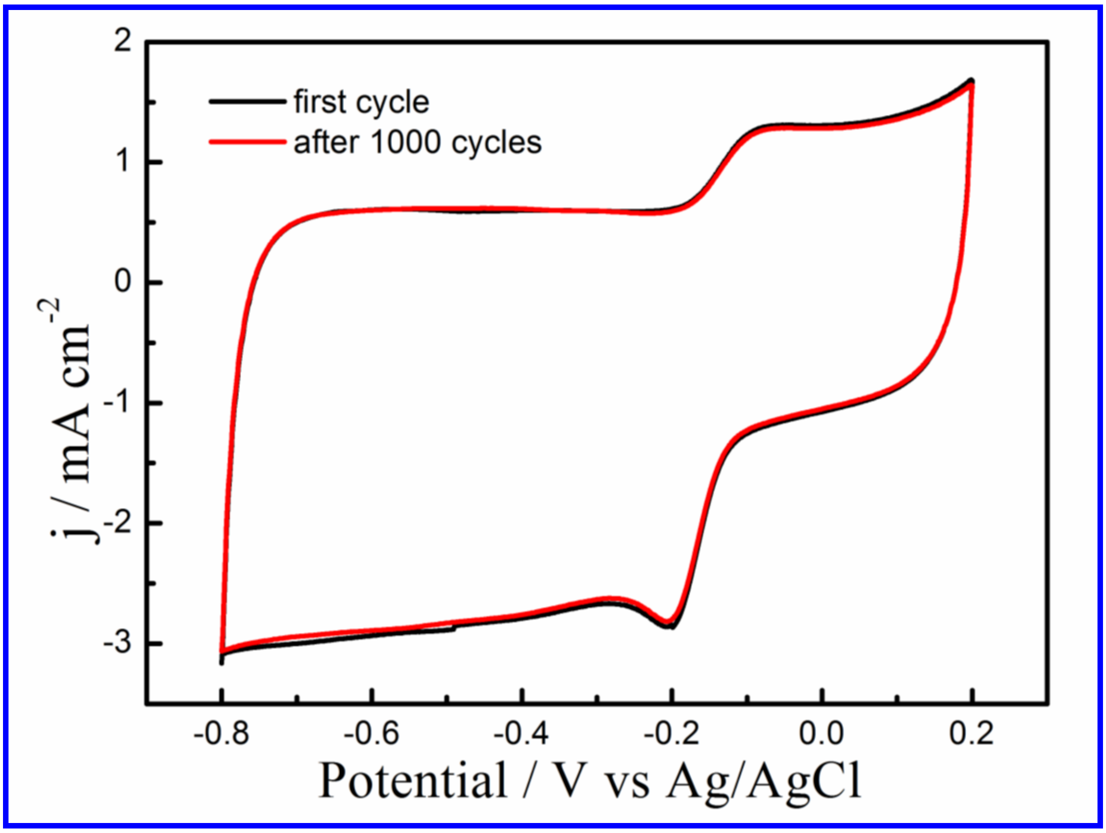

Figure S18. The CVs cycling in O_2 saturated 0.1 M KOH at the scan rate of 50 mV s^{-1}. First cycle (black line) and after 1000 cycles (red line).

Table S1. Comparison of electrochemical performance of NDPC-3-800 with other carbon materials derived from biomass materials.

Carbon precursor	Activator	Surface area (m^2 g^{-1})	Pore volume (cm^3 g^{-1})	Electrolyte	Specific capacitance	Ref.
Poplar catkins	$ZnCl_2$	1463	1.31	1 M H_2SO_4	251 F g^{-1} (0.5 A g^{-1})	1
Hexagonia apiaria	KOH	1280	-	6 M KOH	324 F g^{-1} (1.0 A g^{-1})	2
Soybean Root	KOH	2143	0.94	6 M KOH	268 F g^{-1} (0.5 A g^{-1})	3
Willow catkin	KOH	1533	0.92	6 M KOH	298 F g^{-1} (0.5 A g^{-1})	4
Willow catkins	KOH	1775.7	0.8516	6 M KOH	292 F g^{-1} (1 A g^{-1})	5
Fresh lotus	H_2O_2, HAc	1015	0.824	3 M KOH	340 F g^{-1} (0.5 A g^{-1})	6
Sugar cane bagasse	KOH, Urea	2905.4	2.05	6 M KOH	301.9 F g^{-1} (1.0 A g^{-1})	7
Indicalamus leaves	PTFE	1801	1.45	6 M KOH	326 F g^{-1} (0.5 A g^{-1})	8
Sodium glutamate	NaCl	1007.62	0.56	6 M KOH	320 F g^{-1} (1.0 A g^{-1})	9
Corncob sponge	KOH	1874	0.945	6 M KOH	404 F g^{-1} (0.1 A g^{-1})	10
Pomelo peels	$(NH_4)_2HPO_4$	807.7	0.4378	2 M KOH	240 F g^{-1} (0.5 A g^{-1})	11
Ant powder	KOH	2650	1.4	6 M KOH	576 F g^{-1} (1.0 A g^{-1})	12
Clover stems	KCl	1459	0.912	1 M H_2SO_4	451 F g^{-1} (0.5 A g^{-1})	13
Round-grained rice	-	115	-	6 M KOH	321 F g^{-1} (0.5 A g^{-1})	14
Paulownia sawdust	NaOH	1962	0.94	6 M KOH	227 F g^{-1} (2.0 mV s^{-1})	15
Juncus	$ZnCl_2$	1379.9	1.163	6 M KOH	290.5 F g^{-1} (0.5 A g^{-1})	This work

Table S2. Summary of the ORR performance of carbon materials derived from biomass materials.

Biomass	Activator	Electrolyte	Peak potential	Hetero-atoms	Ref.
Luffa sponge	NH_3	0.1 M KOH	-0.13 V vs Ag/AgCl	N	16
Bamboo fungus	$ZnCl_2$	0.1 M KOH	0.089 V vs Ag/AgCl	N	17
Malachium Aquaticum	-	0.1 M KOH	0.053 V vs RHE	N	18
Platanus	Urea, Boric acid	0.1 M KOH	0.829 V vs RHE	N, B	19
Soybeans	$FeCl_3$	0.1 M KOH	0.690 V vs RHE	Fe, N	20
Corn silk	NH_3, $FeCl_3$	0.1 M KOH	-	N, P, Fe	21
Honeysuckles	-	0.1 M KOH	0.082 V vs Ag/AgCl	N, S	22
Water hyacinth	$ZnCl_2$	0.1 M KOH	0.88 V vs RHE	N	23
Lycium barbarum L	NaCl	0.1 M KOH	-0.22 V vs SCE	N	24
Soybean	NH_3 H_2O	0.1 M KOH	-0.27 V vs SCE	N	25
Cocoon silk	$ZnCl_2$	0.1 M KOH	-0.13 V vs Ag/AgCl	N	26
Pig blood	$FeCl_3$	0.1 M KOH	-0.19 V vs SCE	N	27
Juncus	$ZnCl_2$	0.1 M KOH	-0.173 V vs Ag/AgCl	N	This work

References

1. S. Gao, X. Li, L. Li and X. Wei, *Nano Energy*, 2017, 33, 334-342.
2. L. Deng, W. Zhong, J. Wang, P. Zhang, H. Fang, L. Yao, X. Liu, X. Ren and Y. Li, *Electrochimica Acta*, 2017, 228, 398-406.
3. N. Guo, M. Li, Y. Wang, X. Sun, F. Wang and R. Yang, *Acs Applied Materials & Interfaces*, 2016, 8, 33626-33634.
4. Y. Li, G. Wang, T. Wei, Z. Fan and P. Yan, *Nano Energy*, 2016, 19, 165-175.
5. L. Xie, G. Sun, F. Su, X. Guo, Q. Q. Kong, X. M. Li, X. Huang, L. Wan, W. Song and K. Li, *Journal of Materials Chemistry A*, 2016, 4, 1637-1646.
6. S.-Y. Lu, M. Jin, Y. Zhang, Y.-B. Niu, J.-C. Gao and C. M. Li, *Advanced Energy Materials*, 2017, 1702545.
7. K. Zou, Y. Deng, J. Chen, Y. Qian, Y. Yang, Y. Li and G. Chen, *Journal of Power Sources*, 2018, 378, 579-588.
8. J. Huang, L. Chen, H. Dong, Y. Zeng, H. Hu, M. Zheng, Y. Liu, Y. Xiao and Y. Liang, *Electrochimica Acta*, 2017, 258, 504-511.
9. W. Qian, J. Zhu, Y. Zhang, X. Wu and F. Yan, *Small*, 2015, 11, 4959-4969.
10. Y. Liu, Z. Xiao, Y. Liu and L. Z. Fan, *Journal of Materials Chemistry A*, 2017, 5, 24178–24184.
11. Z. Wang, Y. Tan, Y. Yang, X. Zhao, Y. Liu, L. Niu, B. Tichnell, L. Kong, L. Kang, Z. Liu and F. Ran, *Journal of Power Sources*, 2018, 378, 499-510.
12. G. Zhao, C. Chen, D. Yu, L. Sun, C. Yang, H. Zhang, Y. Sun, F. Besenbacher and M. Yu, *Nano Energy*, 2018, 47, 547-555.
13. C. Wang, D. Wu, H. Wang, Z. Gao, F. Xu and K. Jiang, *Journal of Colloid and Interface Science*, 2018, 523, 133-143.
14. F. Lai, G. Zhou, F. Li, Z. He, D. Yong, W. Bai, Y. Huang, W. W. Tjiu, Y.-E. Miao, B. Pan and T. Liu, *ACS Sustainable Chemistry & Engineering*, 2018, 6, 3143-3153.
15. X. Liu, M. Zheng, Y. Xiao, Y. Yang, L. Yang, Y. Liu, B. Lei, H. Dong, H. Zhang and H. Fu, *Acs Applied Materials & Interfaces*, 2013, 5, 4667-4677.
16. J. Li, S. Wang, Y. Ren, Z. Ren, Y. Qiu and J. Yu, *Electrochimica Acta*, 2014, 149, 56-64.
17. S. Gao, H. Fan and S. Zhang, *Journal of Materials Chemistry A*, 2014, 2, 18263-18270.
18. H. Huang, X. Wei and S. Gao, *Electrochimica Acta*, 2016, 220, 427-435.
19. C. Cao, L. Wei, Q. Zhai, G. Wang and J. Shen, *Electrochimica Acta*, 2017, 249, 328-336.
20. Y. Liu, J. Ruan, S. Sang, Z. Zhou and Q. Wu, *Electrochimica Acta*, 2016, 215, 388-397.
21. W. Wan, Q. Wang, L. Zhang, H. Liang, P. Chen and S. H. Yu, *Journal of Materials Chemistry A*, 2016, 4, 8602-8609.
22. S. Gao, H. Liu, K. Geng and X. Wei, *Nano Energy*, 2015, 12, 785-793.
23. X. Liu, Y. Zhou, W. Zhou, L. Li, S. Huang and S. Chen, *Nanoscale*, 2015, 7, 6136-6142.
24. L.-X. Zuo, W.-J. Wang, R.-B. Song, J.-J. Lv, L.-P. Jiang and J.-J. Zhu, *ACS Sustainable Chemistry & Engineering*, 2017, 5, 10275-10282.
25. G. Lin, R. Ma, Y. Zhou, Q. Liu, X. Dong and J. Wang, *Electrochimica Acta*, 2018, 261, 49-57.
26. P. Fu, L. Zhou, L. Sun, B. Huang and Y. Yuan, RSC Adv, 2017, 7, 13383 -13389.
27. J. Zhang, Q. Li, C. Zhang, L. Mai, M. Pan and S. Mu, *Electrochimica Acta*, 2015, 160, 139-144.
